# Supplementary material for: Comparative quantum-classical dynamics of natural and synthetic molecular rotors show how vibrational synchronization modulates the photoisomerization quantum efficiency
Source: Nat Commun. 2024 Apr 25;15:3499. doi: 10.1038/s41467-024-47477-0 (PMC11045841; doi:10.1038/s41467-024-47477-0)
Supplement: Supplementary file 1 — Supplementary Information [file 41467_2024_47477_MOESM1_ESM.pdf]

Supplementary Information

Comparative Quantum-Classical Dynamics of Natural and Synthetic Molecular Rotors Show How Vibrational Synchronization Modulates the Photoisomerization Quantum Efficiency

Alejandro Blanco-Gonzalez[1], Madushanka Manathunga[1,2], Xuchun Yang[1], Massimo Olivucci[1,3*]

[1]Department of Chemistry, Bowling Green State University, Bowling Green, OH, 43403, US.

[2]Present address: Department of Chemistry and Department of Biochemistry and Molecular Biology, Michigan State University, East Lansing, MI, 48824, US.

[3] Department of Biotechnology, Chemistry and Pharmacy, University of Siena, I-53100, Siena, Italy. *email: molivuc@bgsu.edu.

Section I. Computational Details

rPSB11@Rh QMMM model, population dynamics, and initial conditions. Multiconfigurational quantum chemistry-based QM/MM model construction. A quantum mechanics/molecular mechanics (QM/MM) model of Rh was constructed (Fig S1a) starting from the 2.2 Å resolution crystallographic structure available in the Protein Data Bank (PDB ID 1U19)[1] and following a reported protocol.[2] The atoms of the retinylidene chromophore, NH group and $C_{\varepsilon}H_3$ substituent form the QM layer, which is treated at the CASSCF level of theory with the 6-31G* basis set. The active space involves 12 electrons in 12 orbitals, which comprises the entire π-system of the retinal chromophore moiety and defines the method of multiconfigurational wavefunction. The rest of the protein, comprising the atoms of the $C_{\delta}H_2$ moiety belonging to the Lys256 side-chain, form the MM layer, which is described by a modified AMBER94 force field featuring specific Lys296 side-chain parameters.[3,4] All side-chains and waters with at least one atom within 4 Å from any atom of the retinal chromophore were kept flexible during the trajectory calculations. All remaining atoms were kept frozen. The 200 initial conditions (geometries and velocities) defining the ground state populations at room temperature are generated via a protocol reported earlier.[2] Briefly, 20 ns MM dynamics simulation at 298K was initiated from an S_0

optimized geometry reproducing the wavelength of the corresponding absorption maxima (λ_{max}). Where 200 snapshots (geometries and velocities) were extracted during the dynamics at 100 ps time intervals after a suitable equilibration time, starting from these snapshots, 200 HF/6-31G*/Amber were propagated for 200 fs and then followed by corresponding S_0 2-root-state-average CASSCF/6-31G*/Amber for 50 fs. The 200 geometries and velocities of the final snapshot of the 50 fs propagation are assumed to represent the Boltzmann distribution (to best account for the PES anharmonicity, a Boltzmann or Wigner sampling based on the Hessian Matrix were not considered) and, therefore, the initial conditions for subsequent quantum-classical trajectory computations starting on S_1. All calculations were performed using Molcas/Tinker package.[5,6]

MeO-NAIP@MeOH QMMM model, population dynamics, and initial conditions. The model of MeO-NAIP@MeOH was constructed via classic molecular dynamics equilibration with periodic boundary conditions (Fig S1b). The MeO-NAIP molecule was taken from a previously reported work[7] (as described in ref. 7; this corresponds to the Z diastereoisomer. Therefore, the investigated isomerization process is Z to E. The E to Z process is not investigated here also due to the lack of corresponding experimental studies). The optimized chromophore is then embedded in the center of a cubic box of size 60 x 60 x 60 Å, containing methanol (MeOH) solvent molecules. The chromophore is kept frozen, and the solvent system is minimized at the molecular mechanics (MM) level, using the conjugate-gradient method with periodic boundary conditions. The nuclear charges of the chromophore atoms for this purpose are calculated using the Electrostatic Potential Fitting Method (ESPF) at the MP2/6-31G* level. The binding and van der Waals parameters are taken from the OPLS_aa force field[8]. The minimized chromophore-solvent system is then relaxed using a 5 ns long molecular dynamics (MD) simulation under the isothermal-isobaric ensemble (NPT), set at 1 atm of pressure and 298 K of temperature using the GROMACS software package [9]. During the first 0.3 ns of the simulation, the system gradually thermalizes to 298 K, equilibrates for the next 0.7 ns, and relaxes for the next 4 ns. From this dynamic simulation step, named production, snapshots are extracted every 10 ps, involving a 20-Å solvent layer to complete a full set of 400 configurations to define the hybrid quantum mechanics / molecular mechanics (QM/MM) model. In this model, the chromophore is treated at the QM level using the complete active space self-consistent field (CASSCF) method with a 6-31G* basis set and an active space comprising 12 electrons in 11 orbitals (CASSCF/631-G*/12,11). The solvent molecules, located

at 4 Å from any chromophore atom, are treated at the MM level (OPLS_aa force field) and are allowed to move during the QM/MM molecular dynamics while the rest of the solvent group remains frozen. The QM/MM dynamics are computed following a single-root CASSCF gradient for 200 fs with a time step of 1 fs (41.3 au), and geometries and velocities of the final snapshot are assumed to represent the Boltzmann distribution (to best account for the PES anharmonicity, a Boltzmann or Wigner sampling based on the Hessian Matrix were not considered) and, therefore, the initial conditions for subsequent quantum-classical trajectory computations starting on S_1. All calculations were performed using Molcas/Tinker package.[5,6]

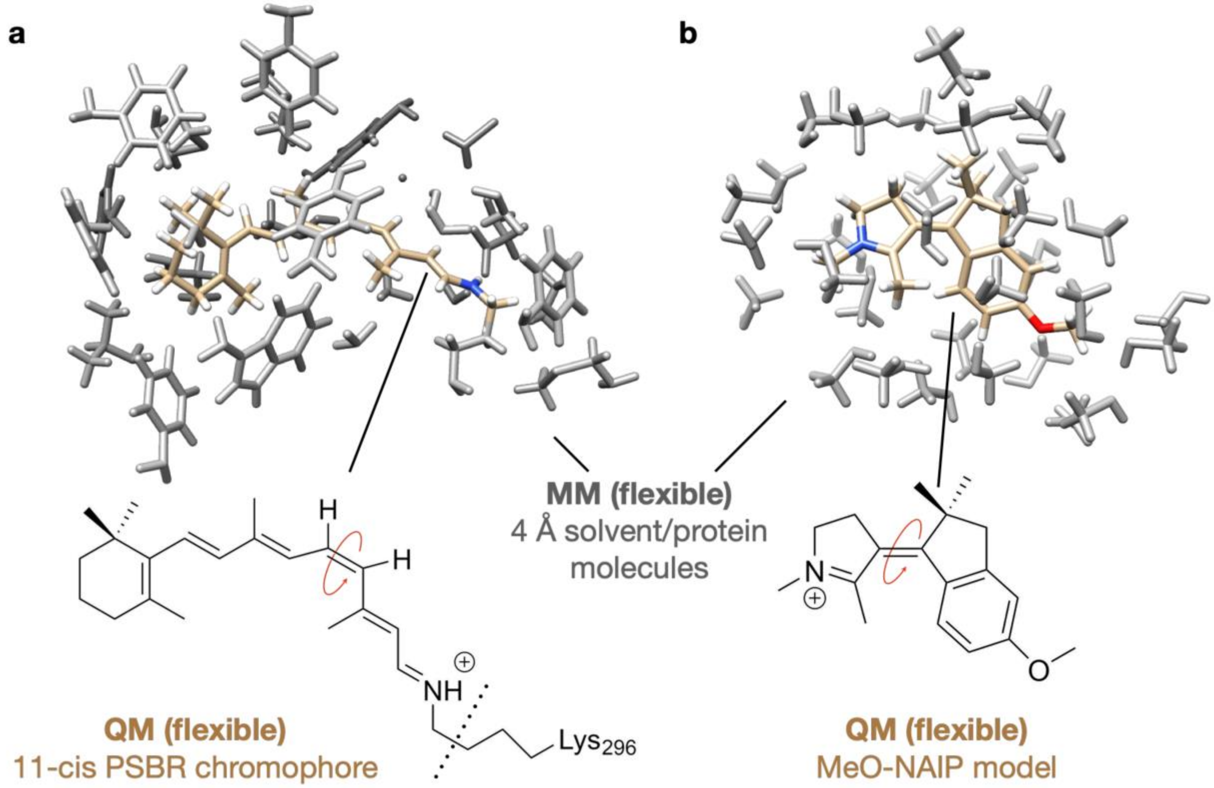

Figure S1. QMMM models of systems under comparison. a) 11-cis PSBR chromophore of Rh, and b) MeO-NAIP (Z diastereoisomer). The red curly arrows indicate the photoisomerization reaction.

rPSB11 and MeO-NAIP isolated model generation. In order to study the protein and survey the induced environmental effects, we repeat the above initial condition calculations for the two isolated chromophores (i.e. in the absence of the protein and methanol solvent for Rh and MeO-

NAIP respectively). Therefore, the sampling is limited to the isolated retinal chromophore with a terminal -C15=N-$C_{\varepsilon}H_3$ group and to the MeO-NAIP cation, corresponding to the same structure employed during solvated study described above, but now in isolated conditions. Therefore, the protocol followed for the initial conditions generations was the same described in the section above but the lack of the environment: protein or methanol, according to each model removes the MM side (protein or solvent atoms treated at MM level) from the previously described QMMM model. In this isolated model, the chromophore is still treated at the QM level using the complete active space self-consistent field (CASSCF) method with a 6-31G* basis set and the corresponding previously reported active space CASSCF/6-31G*. Notice that the MeO-NAIP switch is not a chiral molecule, which means it will create a combination of enantiomers resulting in both clockwise and counterclockwise isomerization, yielding equal statistics.

Final comments on computational methodology. The employed 2-root-state-average CASSCF for Rhodopsin and a 3-root-state-average CASSCF for MeO-NAIP are given for the following reasons: i) These levels reproduce measured dynamics and utilize single-point CASPT2 corrections for measured spectroscopic observables. ii) In the case of Rhodopsin, the introduction of the S_2 in the CASSCF (i.e. S_0, S_1, and S_2 state averaging) calculation makes the S_1 potential energy surface too flat, generating too long excited state lifetimes. The interaction of the S_1 and S_2 states when the retinal is modeled inside the protein environment has been proven to be very limited, demonstrating that S_2 inclusion does not significantly impact the dynamics[10]. iii) In the case of MeO-NAIP, a strategy based on S_0, S_1, and S_2 state averaging had to be applied instead. The reason for this change is that the synthetic system has much higher excitation energies (it absorbs UV-light rather than visible-light) and features a much smaller S_2-S_1 energy gap. In fact, in contrast to Rhodopsin, most of the initial conditions selected to initiate quantum-classical trajectory calculations, at the CASSCF level, show S_2 being the spectroscopic state, and it was therefore necessary to start the trajectory from S_2 rather than S_1. This approach aligns with the protocol benchmarked and adopted in references[11,12]. In essence, we believe a model that captures experimental observables enhances our ability to elucidate reaction mechanisms.

Section II. Excited state lifetime.

The excited-state quantum-classical trajectories were propagated at the 2-root-state-average CASSCF/6-31G*/Amber and 3-root-state-average CASSCF/6-31G*/OPLS-aa level for Rh and MeO-NAIP respectively, following the Tully surface-hop method including the decoherence correction[13,14]. (A test supporting the validity of the Tully surface-hopping method for alkylated or protonated Schiff bases has been recently reported.[15]) The state average was determined according to the values of the oscillator strength presented for each system. All calculations were performed using Molcas/Tinker package.[5,6] In order to determine the S_1 lifetime reported in Table 1, we have used three different approaches: i) following fitting formula (1) comprising a lag time and two exponential decay time, ii) the time what the system decay to half of the S_1 population decay, and iii) average of decay time.

$$f(t) = a_1 e^{(-(\frac{t-t_1}{t_2})^2)} + y_0 + ((1-a_1) - y_0) e^{(\frac{-(t-t_1)}{t_3})} \quad (1)^{16}$$

Section III. QMMM model and population dynamics validation.

The rPSB11@Rh model has been previously validated and consequently referenced in the Table S1 below. This section will focus on the MeO-NAIP@MeOH model, and the validation carried out by predicting the values of a set of properties relevant to the photophysical and photochemical behavior of these systems, which are then compared to the available experimental values. These properties following a comparative aim with the already validated rPSB11@Rh model are:

1) λ_{max} value and corresponding absorption band.
2) Photoproduct appearance time.
3) Excited state lifetime.
4) Excited state vibrational frequencies for δ_{op} and BLA.
5) Quantum efficiency of MeO-NAIP.

Table S1. Comparison between the observed experimental parameters and the computational ones.

rPSB11@Rh	λ_{max} (nm)	product appearance time (fs)	lifetime (fs)	vibrational freq. (cm^{-1}) δ_{op}	vibrational freq. (cm^{-1}) BLA	Φcis-trans %
Obs.	498	< 50[a]	~80[b]	746[a]	1679[a]	67[c]
Comp.	500	> 70[d-i]	98[d-ii] (93[d-iii])	855, 1068[d]	1588[d]	68[d] 72[e] (69[f])

Comp. Isolated	560	-	114[d-ii] (107[d-iii])	-	-	47[d]
MeO-NAIP@MeOH	λ_{max} (nm)	product appearance time (fs)	lifetime (fs)	vibrational freq. (cm^{-1}) δ_{op}	vibrational freq. (cm^{-1}) BLA	Φcis-trans %
Obs.	389[g]	> 300[h]	260[h]	Not-determined	1572[i]	21[j]
Comp.	383	358	281[d-ii] (265[d-iii])	565, 847[d]	1450[d]	30[d]
Comp. Isolated	480	-	254 [d-ii] (240[d-iii])	-	-	55[d]

a. Observed data from ref. [17]. b. Observed data from ref. [18]. c. Observed data from ref. [19]. d. Based on a 200-trajectory ensemble. d-i. Time range from the first successful decay to the first photoproduct with a bathoRh-like/E-isomer absorption. d-ii. Fitted S_1 lifetime of 200 trajectories. d-iii. Average decay time of 200 trajectories. e. Based on a 50-trajectory sample with a Rh model of a full rPSB11 chromophore. f. Based on a 400-trajectory sample with a Rh model of a reduced rPSB11 chromophore from ref. [2]. g. Observed data from ref. [20]. h. Observed data from ref. [21]. i. Observed data from ref. [7]. j. Observed data from ref. [22]

We evaluated the λ_{max} value (property 1) from the absorption band (Fig. S2a) computed at the 3-root-state-average CASPT2//CASSCF/6-31G*/ OPLS_aa level of theory. As reported in Supplementary Table S1, the computed 383 nm value is in close agreement with the observed value of 389 nm. The results of the population dynamics allowed us to predict the rest of the properties analyzed. To compute the "photoproduct appearance time," (property 2) we use both excitation energy as well as geometrical criteria. For the excitation energy, we selected the small group of trajectories reaching the faster $IS_{S1/S0}$ and leading to successful decay events. We define the shortest time employed by the fastest trajectory to match the computed e-isomer photoproduct absorption λ_{max} value as the upper limit to the photoproduct appearance time (Fig. S2b). The trajectory matches the photoproduct absorption value at 348 fs, considering this time as a predicted photoproduct appearance time, which may be compared with the experimental results suggesting photoproduct detection at time scales higher than 300 fs. The e-isomer photoproduct equilibrium structure necessary for the computation of the photoproduct appearance time defined above was generated by performing an S_0 geometry optimization at the CASSCF/6-31G*/OPLS_aa level from one snapshot (geometry) of the trajectories analyzed once the molecule hop and relaxed on S_0 potential surface. The equilibrium e-isomer structure whose λ_{max} value was determined via a vertical excitation energy calculation at the 3-root-state-average CASPT2//CASSCF/6-

31G*/OPLS_aa level presenting a value of 304 nm (94 kcal mol^{-1}). The excited state lifetime (property 3) is computed by following different approaches yielding equivalent results: i) fitting the S_1 population decay with a previously employed Gaussian-plus-exponential equation (1)[16] (Fig. S2c), ii) taking the time at the system decays to half the S_1 population and iii) as an average value of decay time. The predicted 281 fs (from the fitting), 253 fs (from half of the S_1 population), and 265 fs as the average decay time values must be compared with the observed 260 fs value.[2] The S_1 vibrational frequencies (property 4) of the δ_{op} (60 fs and 40 fs periods, equivalent to 565 cm^{-1} and 847 cm^{-1}) and BLA (23 fs period, equivalent to 1450 cm^{-1}) are obtained based on their average progressions (Fig. S2d). These are defined as the numerical average of the corresponding δ_{op} and BLA values at each time point during the excited state progression. There is no experimental value for δ_{op}, but the BLA frequency value compares with the observed values of 21 fs, equivalent to 1572 cm^{-1}. The $\Phi_{cis\text{-}trans}$ (property 5) was calculated as the ratio of the number of trajectories forming the photoproduct (i.e. this is determined by looking at the α value at the last time step) over the total number of decayed trajectories. The predicted and observed values are 0.30 and 0.21, respectively. In conclusion, the values of the "observables" 1-5 predicted based on the MeO-NAIP@MeOH QM/MM model and population dynamics indicate a semi-quantitative agreement with the experimental observations. These properties have been computed, limiting the simulation (mainly due to the high computational cost) to 200 trajectories.

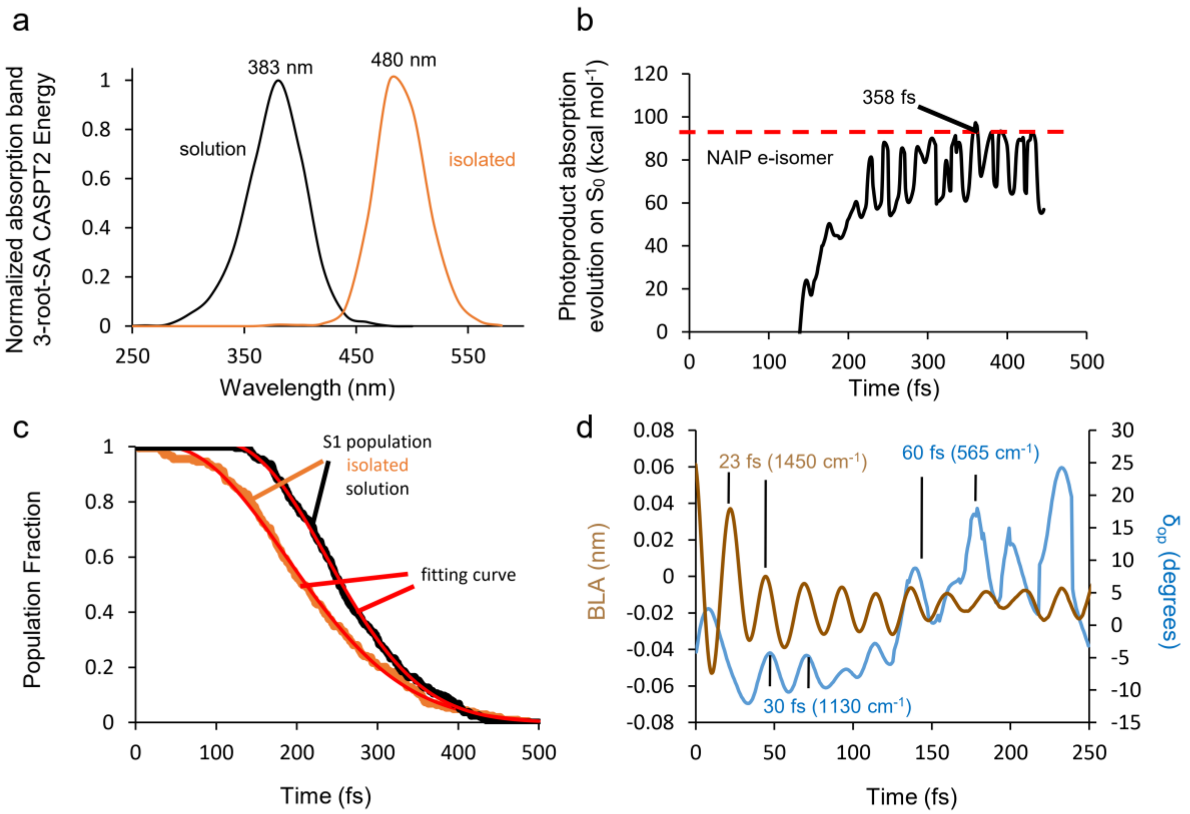

Figure S2. Population dynamics analysis. a) The absorption band is computed to determine λ_{max} value by Gaussian fit of the total population: solution (black curve) and isolated conditions (orange curve). b) Photoproduct (NAIP e-isomer) formation from sub-150 fs fastest decay trajectory. The curves indicate the excitation energy progression, and the dashed red line indicates the S_0-S_1 excitation energy for the fully relaxed NAIP e-isomer structure. c) Excited state lifetime fitting. The S_1 population decay curve in solution (black) and isolated (orange) conditions, with theirs corresponding fitting function (red). d) Average value progression of δ_{op} (in blue) and BLA (in brown) for the S_1 population. FFT analysis is used to determine the frequencies.

Section IV. Statistical analysis

An important part of the present study is the statistical analysis of the rPSB11@Rh and MeO-NAIP@MeOH hop points. Accordingly, the following properties have been determined at each hop point:

A. reactive vs. unreactive hop.

B. negative vs. positive $d\tau/dt$,

C. magnitude of dτ/dt

All velocities are calculated numerically in terms of the differences between the values of successive 1 fs time steps.

We use properties A-C to classify the molecular population in different subsets. Of course, we are mostly interested in the properties of the reactive subpopulations, which are defined as the group of trajectories achieving the product geometry after the hop. These subpopulations are then analyzed based on the corresponding dτ/dt sign and amplitude. As an example, Scheme 1 shows how a sufficient condition (approximate) for reactivity was determined via the analysis illustrated in the left branch of the diagram.

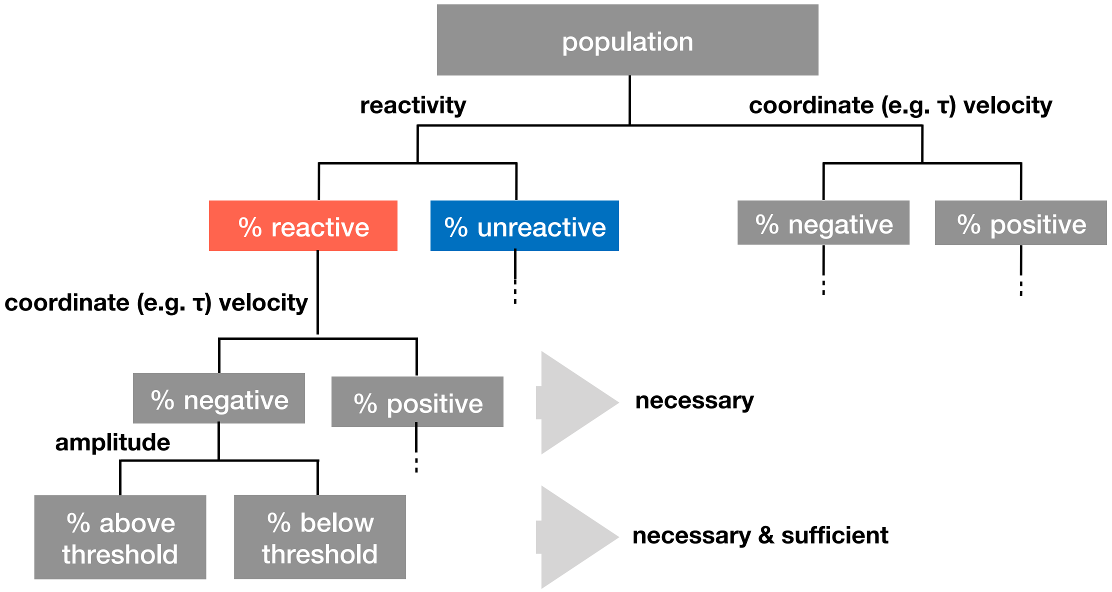

Scheme 1. Analysis of the hop points of the entire population. The left and right branches correspond to analysis carried out according to reactivity (left) or geometrical (right) criteria. In the left part of the diagram, the reactive subpopulation (red box) is analyzed to find out, for instance, if all trajectories in the subpopulation have $d\tau/dt^{decay} < 0$.

A $d\tau/dt^{decay} < 0$ is not a *sufficient* condition for reactivity. As discussed in the main text, the populations of reactive trajectories for the rPSB11@Rh and MeO-NAIP@MeOH systems are

directly associated with $d\tau/dt^{decay} < 0$, with a 15% tolerance for the validity of this relationship. Similarly, we can establish the relationship between unreactive trajectories and $d\tau/dt^{decay} > 0$. This relationship is supported by the relatively large percentages (81% and 92% for the natural and synthetic motors, respectively) of trajectories with $d\tau/dt^{decay} > 0$ that are indeed unreactive in solvated conditions. However, as we discussed in the main text, these relationships are not equally valid for the artificial system. In other words, most of the reactive trajectories have $d\tau/dt^{decay} < 0$, but not all the population fractions with $d\tau/dt^{decay} < 0$ react, as shown in Table S2. Specifically, only 52% of the trajectories with $d\tau/dt^{decay} < 0$ are reactive, and therefore not all the unreactive trajectories have $d\tau/dt^{decay} > 0$.

Table S2. Analysis of the population reactivity. The percentages correspond to rPSB11@Rh and MeO-NAIP@MeOH population fractions satisfying the criteria given on the left entry. The values in square brackets refer to the isolated chromophores.

	population fraction with $d\tau/dt^{decay} > 0$[1]		population fraction with $d\tau/dt^{decay} < 0$[1]	
	rPSB11@Rh	MeO-NAIP@MeOH	rPSB11@Rh	MeO-NAIP@MeOH
	%	%	%	%
Reactive[2]	19 [25]	8 [61]	85 [66]	52 [62]
Unreactive[2]	81 [75]	92 [39]	15 [34]	48 [38]

1. % with respect to the full $d\tau/dt^{decay} > 0$ or $d\tau/dt^{decay} < 0$ population.
2. reactivity criterium defined looking at the S_0 configuration reached after the hop.

Section V. A promoter modulates the dτ/dt amplitude.

We construct a basic model of the average progression of the populations by assuming that α has two components, α_I and α_{II}, associated with a monotonic progression towards $IS_{S1/S0}$ and with the δ_{op}-coupled oscillatory motion, respectively (see Fig. S3a, top-left).

Consistently with trajectory visualizations, $\alpha_{II} - 0.5\delta_{op}$ describes a periodic out-of-phase change in pyramidalization, namely a change from sp^2 to sp^3 hybridization and back, at C2 and C3 (see Fig. S3a, top-right) that can also be described as an R1, R4 wag coupled with a C1, C4 wag. In our approximate model of the average progression, α_I represents the isomerization mode, and $d\alpha_I/dt$ is considered constant, while $d\alpha_{II}/dt$ and $d\delta_{op}/dt$ are harmonic functions characterized by phase, period, and amplitude. The $d\alpha_I/dt$ value and the $d\alpha_{II}/dt$ parameters for rPSB11@Rh and MeO-NAIP@MeOH are obtained by fitting the average $d\alpha/dt$ value in Fig. S3b top. The $-0.5d\delta_{op}/dt$

parameters are instead obtained by fitting the average values of Fig. S3b bottom. The results of the fitting are graphically displayed in Fig. S3c.

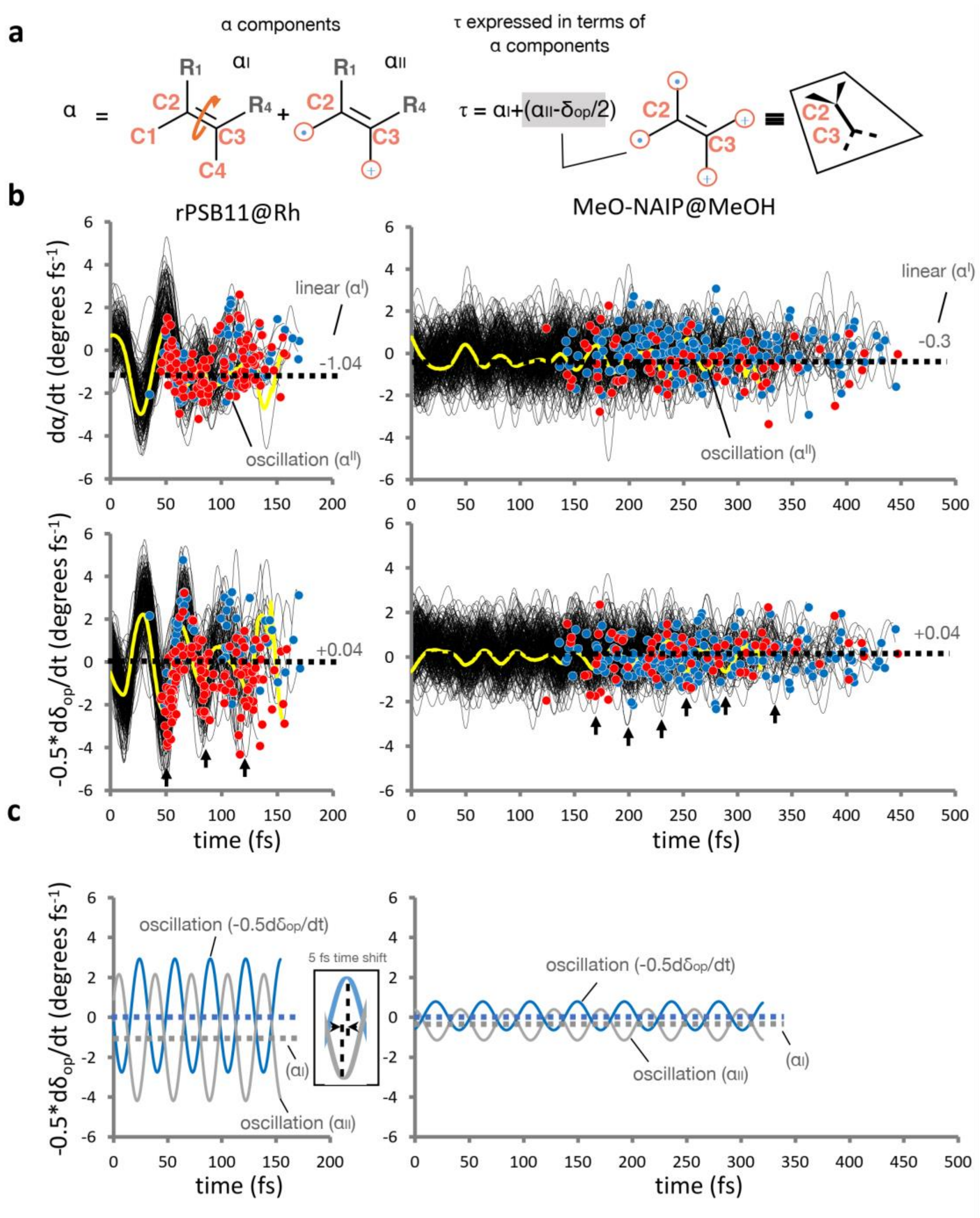

Figure S3. Analysis of the average α and δ_{op} progressions. a) Left. Schematic representation of the linear (α_I) and a harmonic (α_{II}) component of α skeletal twist (or wag). Right. Model expression of τ expressed in terms of α components and schematic representation of its total oscillatory component. b) Top, progression

of α for rPSB11@Rh (left) and MeO-NAIP@MeOH (right). The black lines represent the population motion along S_1 before the decay point (represented by red and blue circles). The yellow curves follow the average value. The horizontal dashed line corresponds to the constant $d\alpha_I/dt$ value. Bottom, same as the top but for the oscillatory component $-0.5d\delta_{op}/dt$. c) Modelled velocities of the oscillatory τ component $d\alpha_{II}/dt$ and $-0.5d\delta_{op}/dt$.

According to our model, the average overlap velocity is $d\tau/dt = d\alpha_I/dt + d(\alpha_{II} - 0.5\delta_{op})/dt$. This expression points to two effects impacting the population fraction with $d\tau/dt^{decay} < 0$. The first operates in systems with a *short IDT and narrow DTL* (e.g. rPSB11@Rh) where the isomerization velocity $d\alpha_I/dt$ allows the center of a *compact* population to enter the $IS_{S1/S0}$ and decay on a timescale of half $\alpha_{II} - 0.5\delta_{op}$ period. In these systems, the phase of $d(\alpha_{II}-0.5\delta_{op})/dt$ (i.e. the direction of the pyramidalization velocity) at decay determines the $d\tau/dt^{decay} < 0$ fraction. This is illustrated in Fig. S4a, where two compact sets of trajectories reach the decay region at different times. If at decay, $d(\alpha_{II} - 0.5\delta_{op})/dt$ is negative (i.e. $\alpha_{II} - 0.5\delta_{op}$ is decreasing), then $d\tau/dt^{decay} < 0$. In other words, during a fast CCW isomerization where $d\alpha_I/dt < 0$, the $d\tau/dt^{decay} < 0$ fraction is maximized when $d(\alpha_{II} - 0.5\delta_{op})/dt < 0$ and minimized when $d(\alpha_{II} - 0.5\delta_{op})/dt > 0$ at the point of decay. Thus, the pyramidalization velocity *promotes* the power-stroke that, when synchronized with $d\alpha_I/dt$, *will periodically enhance the absolute amplitude of $d\tau/dt^{decay} < 0$.* We call $\alpha_{II} - 0.5\delta_{op}$ the power-stroke promoter or, simply, promoter.

The second effect occurs in systems with long IDT and, therefore, large DTL corresponding to a broad S_1 decay and wide distribution of low amplitude $d\alpha_I/dt < 0$ values at decay (e.g. as in MeO-NAIP@MeOH). The decay of such a diffuse population would span several $\alpha_{II} - 0.5\delta_{op}$ oscillations (Fig. S4b), leading to a decay pattern displaying multiple alternate positive and negative $d(\alpha_{II} - 0.5\delta_{op})/dt$ fractions thus leading to a total $d\tau/dt^{decay} < 0$ fraction approaching 50%.

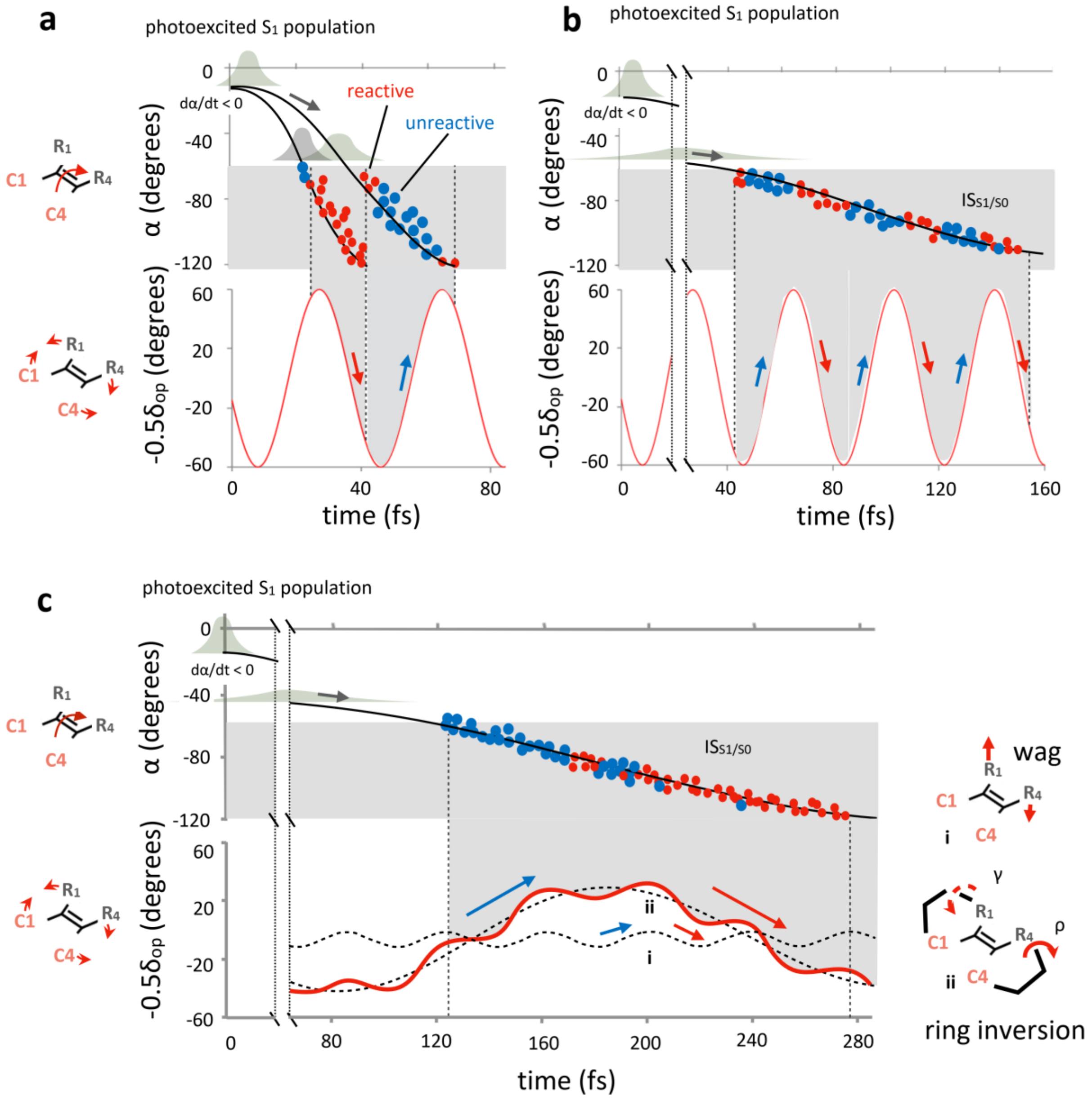

Figure S4. Progression of the monotonic (quasi-linear) and oscillatory component of τ. A replica of Figure 6 from the main text with reordered panels to better explain the basic model. a) Top, schematic representation of the α_I progression and decay associated with two fast-reacting populations (represented by Gaussian profiles). The dark gray area corresponds to the intersection space or decay region ($IS_{S1/S0}$). The red circles represent reactive decay events while the blue circles represent unreactive events. Bottom, corresponding time progression of the α_{II} - 0.5δ_{op} mode. The diagram shows that the two fast populations decay in correspondence of different phases of α_{II} - 0.5δ_{op}. The negative phase corresponding to the fastest population leads to large (left) $d\tau/dt^{decay} < 0$ fraction while the slightly slower population leads to a decreased

$d\tau/dt^{decay} < 0$ fraction (actually a large $d\tau/dt^{decay} > 0$ fraction). b) Same as previous panel but for a population decaying slowly. The corresponding wide distribution of α_I velocities leads to a population decay through multiple α_{II} - $0.5\delta_{op}$ phase variations leading to multiple enhancement and decrease of the produce $d\tau/dt^{decay} < 0$ population fraction. c) Same as previous panel but for an oscillatory component resulting from the superposition of two modes called i and ii (see dashed curves): one high frequency (similar to the oscillatory component in panels a) and b) but lower amplitude mode and one low frequency but higher amplitude (e.g. resulting from an S_1 ring-inversion motion ρ) respectively. A wide distribution of α_I velocities leads to a population decay recovering the characteristics seen in panel a where the population can be divided in fractions decaying with different phases of the coupled oscillatory motion. Again, the fraction decaying with a negative oscillation phase leads to a $d\tau/dt^{decay} < 0$ and therefore to reactive decays.

In the main text, we use the results of the above model to discuss the mechanism of Φ^{iso} modulation. However, for convenience, there we assume, both for each trajectory and for the population (average) values, a proportionality between the directly computable $d\alpha/dt$ and $d\alpha_I/dt$ (i.e. we retain only its monotonic, not oscillatory character) and between $-0.5d\delta_{op}/dt$ and $d(\alpha_{II} - 0.5\delta_{op})/dt$ (i.e. ignoring the α oscillatory character).

Model details. In our approximate model of the average progression, α_I represents the isomerization mode, and $d\alpha_I/dt$ is considered constant, while $d\alpha_{II}/dt$ and $d\delta_{op}/dt$ are harmonic functions (2) characterized by phase (*b*), period (*T*), amplitude (*A*), and vertical shift (*c*).

$$y = Asin\left[\frac{2\pi}{T}(x+b)\right] + c \qquad (2)$$

The $d\alpha_I/dt$ parameters are determined by approximating the α_I curve to a straight line, justified by the quasi-linear monotonic progression towards $IS_{S1/S0}$ that it describes in both systems. Therefore, $d\alpha_I/dt$ are assumed to be constant, and their values are taken from the linear equation's slope. The $d\alpha_{II}/dt$ parameters are obtained by fitting the computed average values of $d\alpha/dt$ (yellow curve in Fig. S3b top). The $-0.5d\delta_{op}/dt$ parameters are instead obtained by fitting the average values (yellow curve in Fig. S3b bottom). The resulting $d\alpha_I/dt$, $d\alpha_{II}/dt$, and $-0.5d\delta_{op}/dt$ models are graphically displayed in Fig. S5a, and once applied $d(\alpha_I + \alpha_{II} - 0.5\delta_{op})/dt$, the resulting curve corresponds to $d\tau/dt$. The result of such calculation is displayed in Fig. S5b top and can be compared with the

computationally derived average dτ/dt (yellow curves) in Fig. S5b bottom. Table S3 shows all the model's parameters.

Table S3. Harmonic model parameters are obtained by fitting the computed average values of dα/dt and -0.5dδ_{op}/dt, while dα_I/dt is assumed to be constant and extracted from the slope of a linear equation.

parameters<br>dτ/dt -model	rPSB11@Rh		MeO-NAIP@MeOH	
	dα_{II}/dt	-0.5dδ_{op}/dt	dα_{II}/dt	-0.5dδ_{op}/dt
A(°/fs)	3.17	2.85	0.774	-0.715
T (fs)	33.5	32.7	43.0	43.4
b (fs)	0.648	3.27	1.75	1.96
c (°/fs)	-1.02	0.0877	-0.379	0.0717
dα_I/dt -constant (°/fs)	-0.566 (from α_I linear fitting<br>y = -0.566x - 13.2)		-0.195 (from α_I linear fitting<br>y = -0.195x - 23.3)	

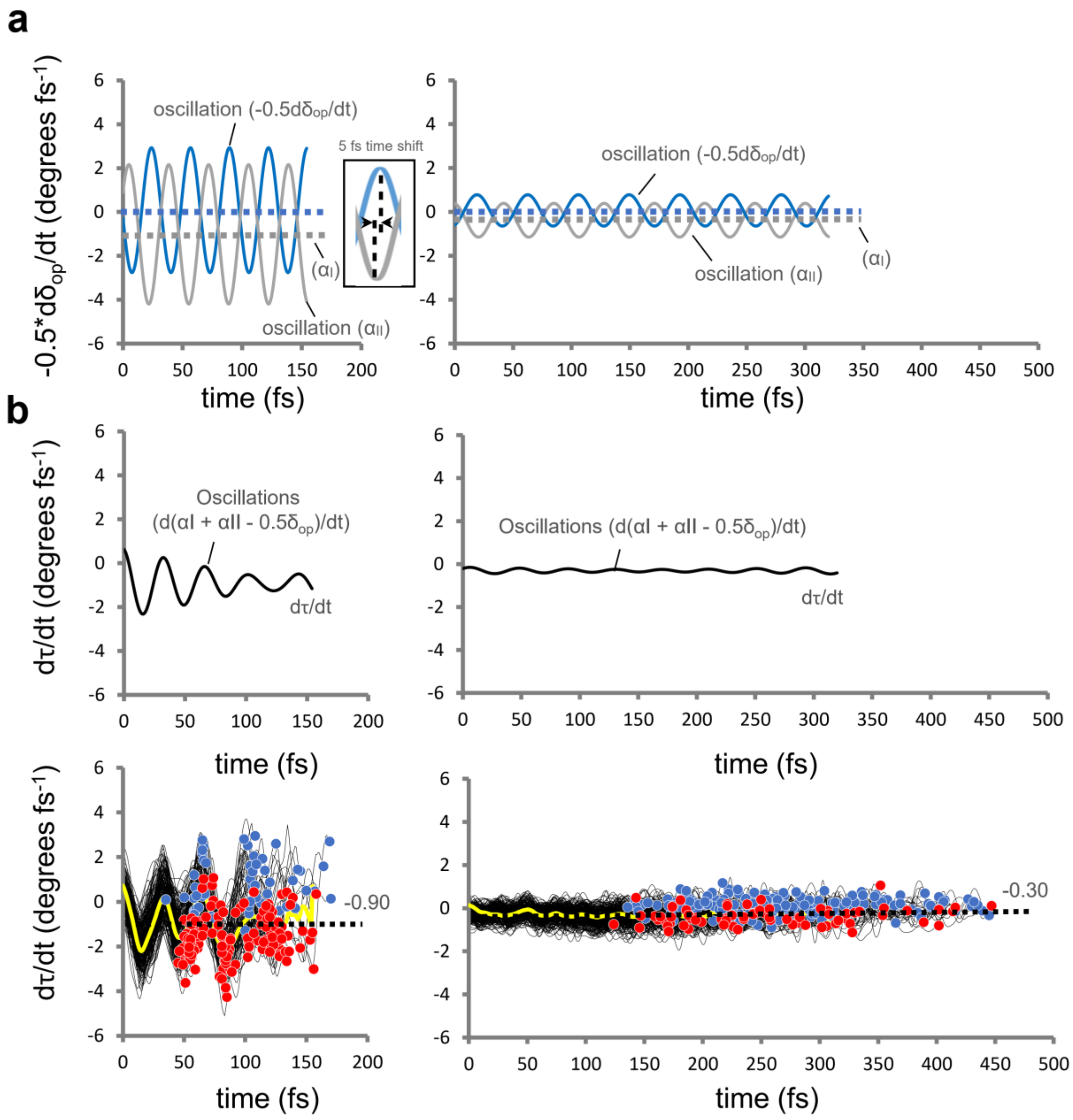

Figure S5. Analysis of the average of dτ/dt progressions. a) Modelled velocities of the oscillatory τ component $d\alpha_{II}/dt$ and $-0.5d\delta_{op}/dt$. b) Top, resulting dτ/dt model. Bottom, progression of dτ/dt for rPSB11@Rh (left) and MeO-NAIP@MeOH (right). The black lines represent the population motion along S_1 before the decay point (represented by red and blue circles). The yellow curves follow the average value. The horizontal dashed line corresponds to the constant $d\alpha_I/dt$ value.

Section VI. Impact of the (catalytic) molecular environment.

Based on Fig. S6 (and Fig. 5 d and e, main text), it is evident that the pyrroline ring oscillates between upward and downward inversions in isolated conditions. In contrast, the same ring adopts a planar configuration in solution, and the ring-inversion is much slower. One hypothesis is that this behavior is dependent on the distinct electronic structures of the ring in the two environments. In the presence of a polar solvent, the S_1 electronic structure of the pyrroline ring stiffens the ring, significantly increasing the ring-inversion barrier and limiting its oscillations. However, this stiffening effect does not occur in isolated conditions, and the ring becomes flexible, oscillating between half-chair-type conformations (out-of-plane). The molecule's electronic structure and corresponding bond lengths appear to explain why the pyrroline ring is rigid in one environment but not in another.

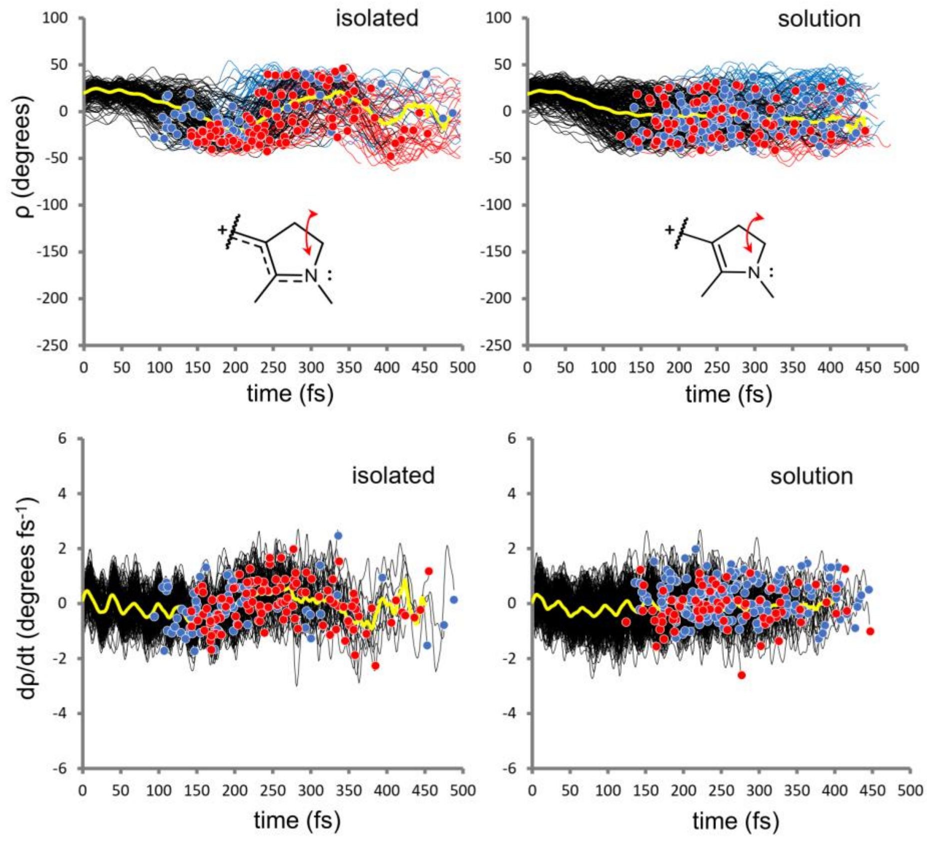

Figure S6. Comparison of the ring-inversion evolution along dynamics of the isolated and solvated MeO-NAIP chromophore. The decay points for reactive and unreactive trajectories are marked with red and blue circles respectively. a) ρ evolution (dihedral). b) dρ/dt evolution (dihedral velocity).

The variation in the amount of positive charge of the pyrroline moiety along some random trajectories was followed in both environments (see Fig. S7). The corresponding charge plots on S_1 reveal distinct behaviors of the isolated (orange) and solution (black) environments, which support the mechanistic hypothesis discussed above and the possible impact on the isomerization quantum efficiency. Firstly, at the beginning (0 fs) of the dynamics, the S_1 positive charge of the solvated system is found to reside almost entirely on the pyrroline moiety, as expected for a system with a counterion (this is a virtual counterion formed by the oriented dipoles associated to the solvent methanol molecules) that keeps the charge close to the N atom. In contrast, the isolated system shows a more distributed charge on S_1 (~0.5 on the pyrroline moiety), corresponding to a delocalized electronic structure connected with the absence of a counterion. Secondly, the low-frequency oscillation observed in the pyrroline moiety charge during the S_1 dynamics appears to be linked to a periodic ring-inversion motion. Notably, the pyrroline moiety charge evolution appears to complete one full oscillation for the solvated system (black) and two oscillations (i.e. corresponding to a ca. halved period) for the isolated system, possibly reflecting the level of stiffness imposed by the electronic configuration over the ring-inversion motion. In this sense a larger frequency would indicate the presence of a stiffer approximately planar ring (see also below).

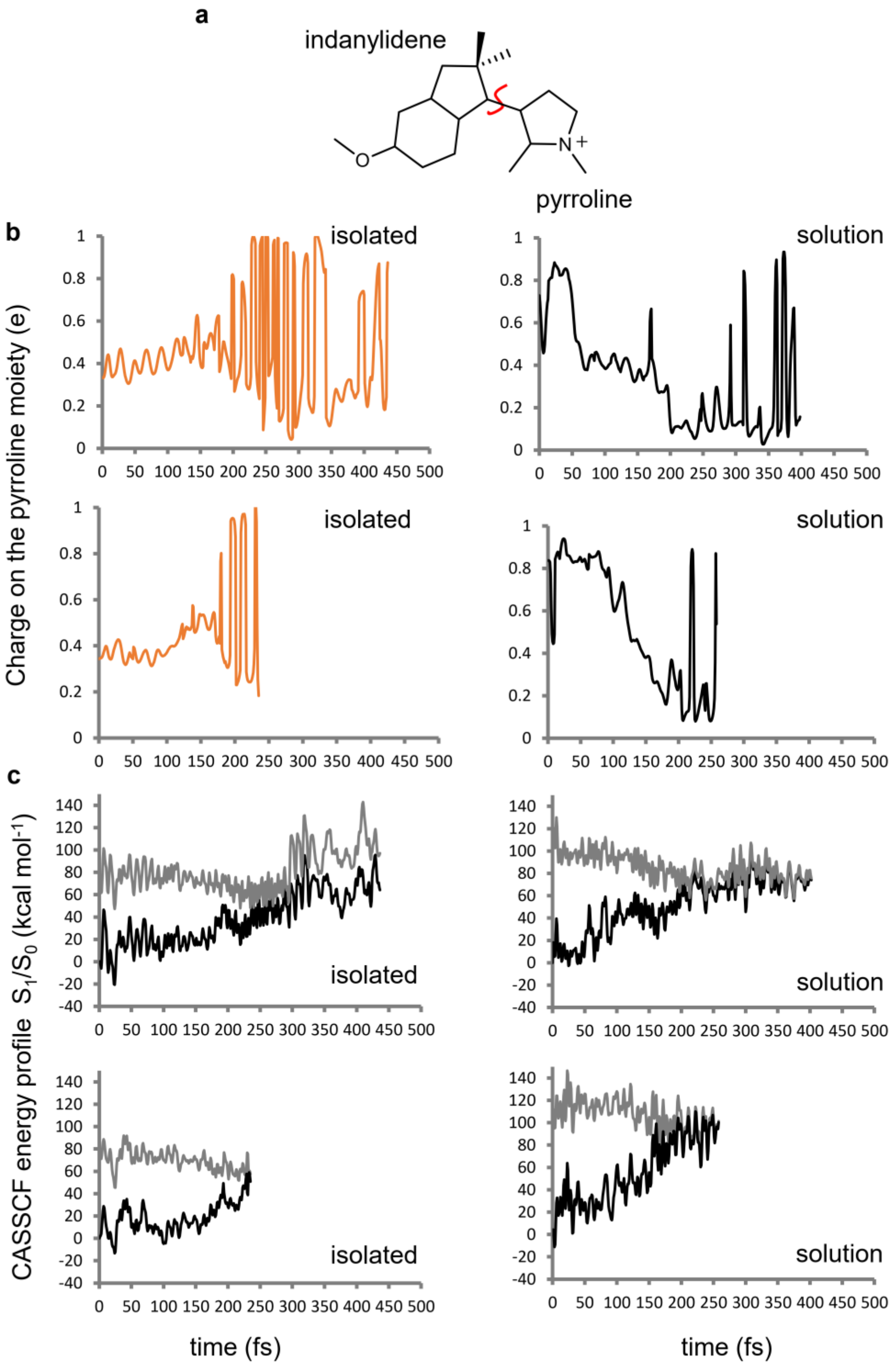

Figure S7. Charge distribution and energy profile of MeO-NAIP. a) MeO-NAIP structure is divided into two moieties: indanylidene and pyrroline. b) Distribution of the positive charge of the pyrroline moiety

along the S_1 for two random trajectories for the two environments: isolated (orange), and solution (black). **C.** CASSCF energy profile for these random trajectories.

To better understand how the electronic structure affects the mechanical (i.e. the low-frequency vibrations such as the ring-inversion) behavior of the pyrroline ring, we have modeled and studied two S_0 model systems that "mimic" different charge distributions on the pyrroline moiety (see Figure 5h, main text). More specifically, such model systems are assumed to represent the different types of electronic configurations observed in isolated and solvated conditions during the S_1 dynamics of MeO-NAIP and, because of that, help to computationally determine whether the dynamic and shape of the potential energy surface associated with a specific electronic structure is consistent with the S_1 dynamics observed for MeO-NAIP. The two selected model systems are:

i. a neutral enamine, that mimics a pyrroline moiety featuring a sp^3 N atom and a localized double bond in position C3=C4 hypothesized to be the dominant electronic structure of a solvated MeO-NAIP in the S_1 state. Consistently with the plot in Fig. 5g bottom, in the main text, such an electronic structure is the one generated via the large charge-transfer motion occurring upon S_1 relaxation in the solvated rotor.
ii. a cyanine, mimics a partially positive charged pyrroline moiety featuring highly delocalized N=C2 and C3=C4 double bonds consistently with the charge plot in Fig. 5g top, main text, and the ring-puckering progression for an isolated MeO-NAIP in the S_1 state in Fig. S8.

The S_0 structures of the models were optimized at the MP2 level and then subjected to 200 fs dynamics at the Hartree-Fock level to extract the final momenta and positions. These were used as starting points for 500 fs S_0 classical trajectory computed at the CASSCF level (in all cases the 6-31G* basis was employed) in isolated conditions. The active space comprises 6 electrons in 5 π-orbitals,

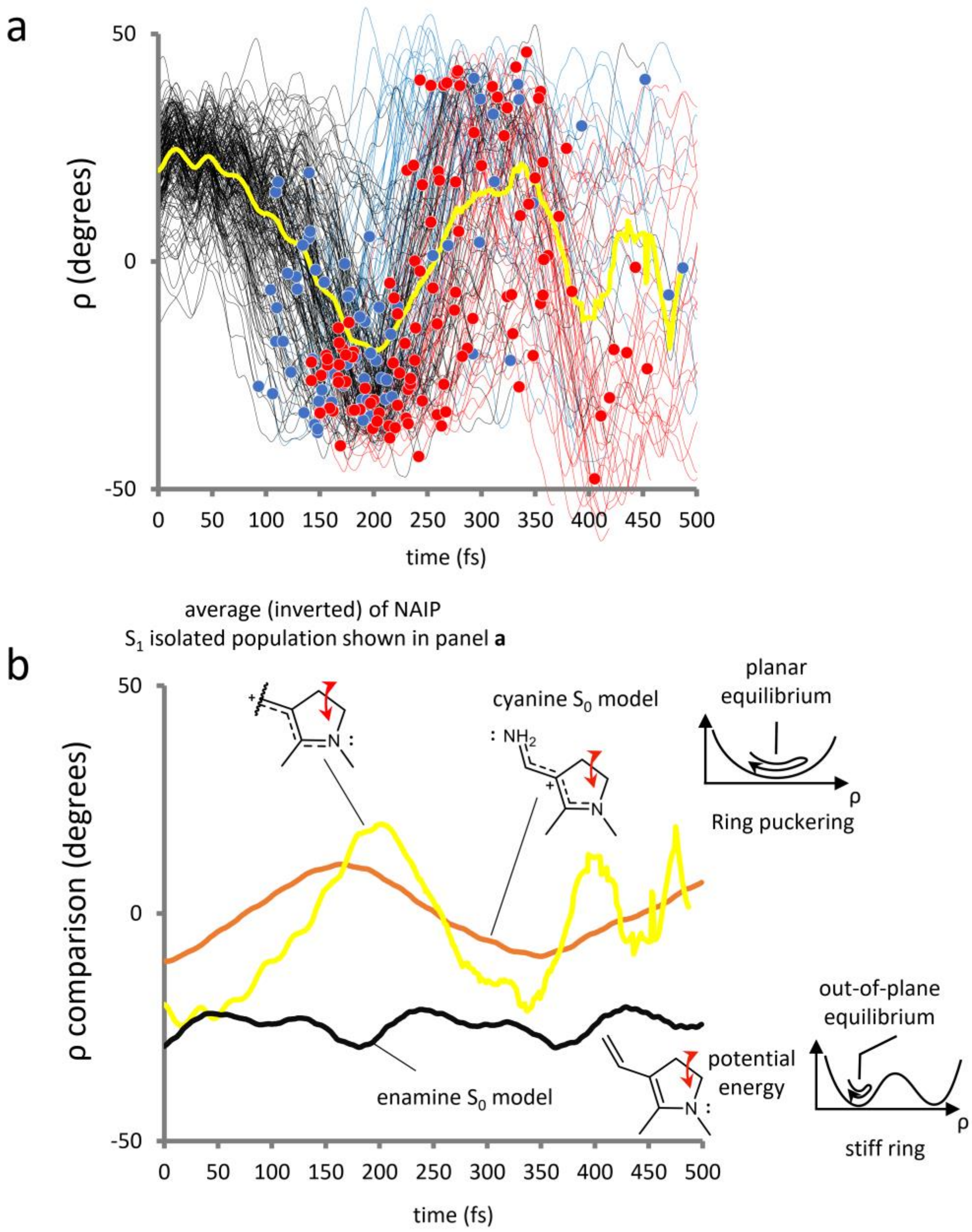

Figure S8. Study of the model systems. a) Time progression of dρ/dt (i.e. pyrroline moiety ring-puckering of MeO-NAIP. b) Comparison between the average time progression of the ring-inversion angle (ρ) (yellow curve inverted from Fig. S8a to match the CW rotation of the models) observed during the S_1 dynamics of MeO-NAIP isolated and the S_0 dynamics of two models: enamine (black) and cyanine (orange).

To gain a better understanding of the relationship between ring-inversion and electronic structure, we have investigated the shape of the S_0 potential energy surface along the out-of-plane ring distortion. This required the identification of two minima corresponding to inverted ring

conformations (half-chair and inverted half-chair) and the transition state (TS) connecting them, which defines the energy barrier that must be overcome for the ring-inversion. The hypothesis is that such energy barrier, if it exists, may restrain the pyrroline moiety oscillatory motion which would instead require, to promptly oscillate, a single potential energy wall with a suitable curvature.

Table S4 summarizes the results of the energy profile mapping along the ring-inversion coordinate that started by determining the conformational minima involved in the ring-inversion and their transition states (TS). To identify the TS structure, we initiated a search for the saddle point in the S_0 PES and a subsequent optimization of the structure found. We then conducted a verification step to confirm that the TS describes the reaction in question, which included hessian calculation to find the imaginary frequency and the corresponding reaction vector connecting the two conformational minima. All optimizations were performed at the 3-root-CASSCF level in the gas-phase for consistency with the MeO-NAIP studies.

Table S4. Relative CASSCF energy (with respect to the lowest minimum) and ring-inversion angle for the conformational minima and TS structures of the three model systems studied.

	enamine		cyanine	
	Energy (kcal mol^{-1})	ρ (degrees)	Energy (kcal mol^{-1})	ρ (degrees)
Min 1	0.0	-25.0	0.0	2.3
TS	4.0	0.1	-	-
Min 2	0.0	24.6	0.0	-0.1

Table S4 shows that the only model with well-defined conformational minima is the enamine model. These minima are not planar and are separated by a ring-inversion energy barrier of about 4 kcal mol^{-1} (as schematically shown in Fig. S8b and Fig. S9). In fact, the located minima correspond to mirror images displaying a pyramidalized (sp^3) nitrogen with an inverted configuration in the pyrroline moiety. The TS connecting these minima is, therefore, planar. In contrast, the cyanine model has nearly planar conformational minima, which means this model displays, substantially, a single conformer located at the center of a relatively shallow potential wall (see Fig. S8b). These models are two cases with the charge translocated (enamine) and with the charge strongly delocalized (cyanine). In the cyanine, where the N atom is sp2, the planar ring

must find itself at the center of shallow energy profiles along the ring-inversion motion, allowing the system to oscillate around a planar configuration. While in the enamine (sp^3 hybridization), the system has, hypothetically, a lesser propensity to planarize the pyrroline moiety leading to a higher oscillatory frequency around a very distorted ring value. The simulation indicates the existence of a different potential energy valley for each model, as indicated in Fig. S8b.

The frequency of the ring-inversion mode was determined by computing the Hessian at the minimum geometry for each model. The resulting frequencies were 174 cm^{-1} (enamine), and 80 cm^{-1} (cyanine). These frequencies agree with the observed dynamics for the models (see Fig. S8b). The enamine model completed three oscillations at a higher frequency with limited amplitude due to a 4 kcal mol^{-1} barrier, while the cyanine model completed one full oscillation at lower frequency.

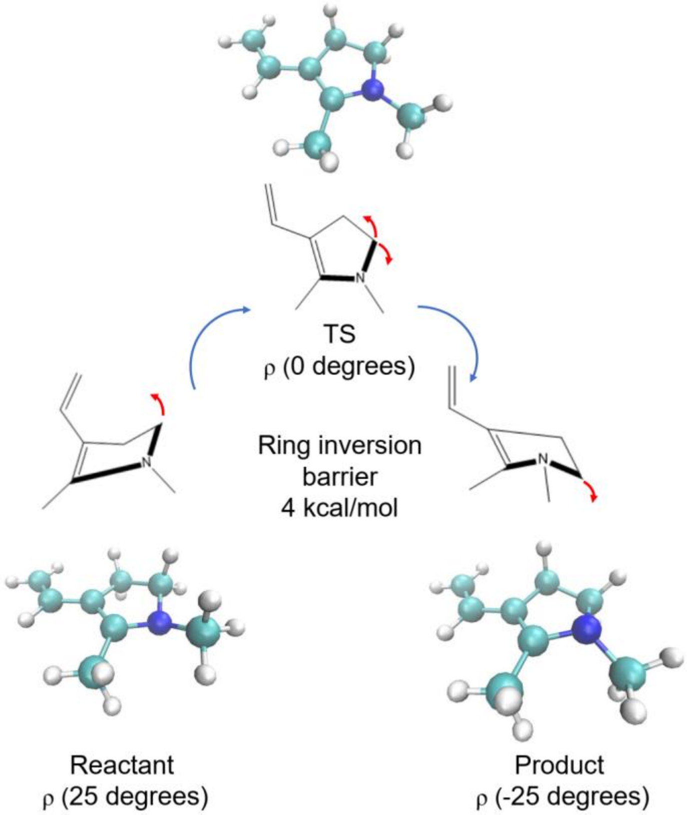

Figure S9. Enamine model TS and minima structures involved in the ring-inversion process. The optimized TS structure corresponds to a planar configuration of the ring and the reaction vector extracted from this calculation allowed to verify that the TS structure is the one connecting the two minima (reactant and product) with two half-chair configurations up and down of the ring. The ring-inversion energy barrier was determined at 4 kcal mol^{-1} over the minima.

A further test was carried out to see if the Φ^{iso} inhibitory effect observed for MeO-NAIP in methanol is exclusively due to electronic reasons (as concluded above) or if additional effects (e.g., hydrogen bonding, steric hindrance, or others) brought up by a different environment is modulating the promoter motion. Accordingly, we constructed a model of MeO-NAIP in the aprotic polar solvent dimethyl sulfoxide (DMSO) following the same protocol already described for methanol solvent to evaluate the ring-puckering ρ mechanism in a different environment and extract some valuable conclusions.

We evaluated the ρ evolution along the S_1 population dynamic (500 fs) represented by 100 trajectories of the resulting MeO-NAIP@DMSO model and compared it with the population dynamics reported for isolated MeO-NAIP and MeO-NAIP@MeOH (see Figure S10). The results show that both polar solvents quench the ring-puckering motion with respect to isolated conditions. However, the population fraction that overpasses the planarity energy barrier and produces a ring oscillation between positive and negative ρ values is slightly higher in DMSO (34%) than in MeOH (26%). Notice that the population fraction is selected based on trajectories that complete one oscillation within the initial ca. 250 fs which also coincides with the S_1 lifetime (at least in isolated condition and MeOH). As discussed above, this quenched behavior in MeOH correlates with a reduction in quantum yield (Φ^{iso}), as it limits the fraction of the population capable of achieving significant ring distortion necessary to enhance the magnitude of dτ/dt velocity. The Φ^{iso} evaluation in DMSO is not discussed in the present study since this environment dramatically changes the excited state lifetime (beyond 500 fs) due to an interaction between bounded (S_2) and unbounded (S_1) electronic states limiting the number of MeO-NAIP@DMSO trajectories that decay to S_0. The investigation of the DMSO must be continued but due to the high computational cost could not be completed within the scope of the present study. Although we cannot correlate the higher percentage of the fraction conducting to oscillation with Φ^{iso} in DMSO, our MeO-NAIP@DMSO simulation demonstrates the potential for ring-inversion in each environment with very different features (volume and viscosity).

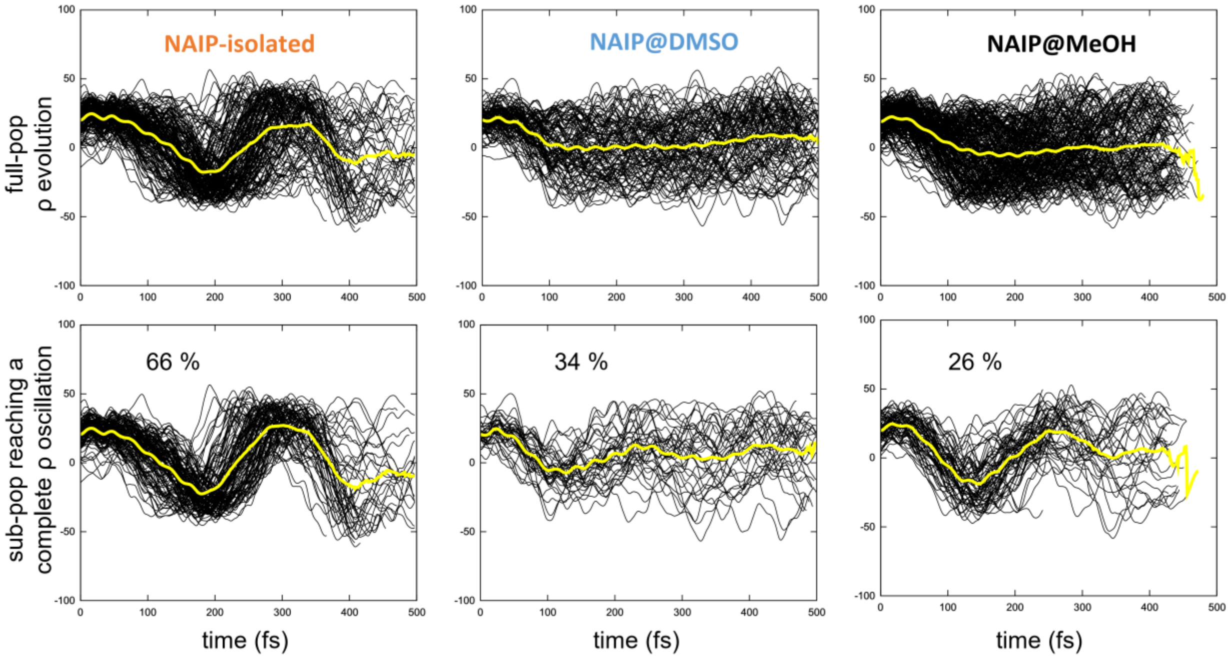

Figure S10. Evolution of the ρ angle across various environments. The top panel illustrates the full population dynamics, while the bottom panel highlights the percentage of sub-population fraction. achieving a complete oscillation within the initial ca. 250 fs coincides with the S_1 lifetime and the intrinsic period of ρ. Black trajectories denote the population motion, and the yellow curves follow the average value.

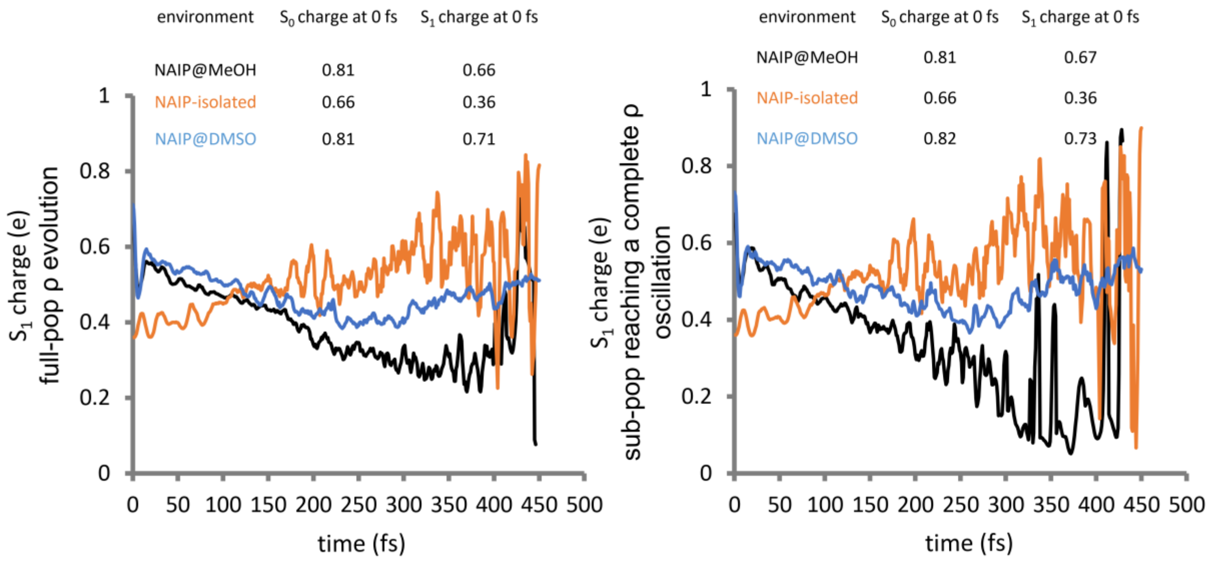

Figure S11. Time progression of the average S_1 charge on the pyrrolinium moiety of MeO-NAIP in three different environments and along the same trajectory set of (left) Figure S10, top panels, and (right) Figure S10, bottom panels.

The analysis presented above is further validated by charge distribution studies of the pyrroline moiety on the S_1 state (see Figure S11). This confirms the dominant influence of electronic factors in governing the ring-puckering phenomena quenched in polar solvents. Collectively, our findings elucidate the complex interaction between molecular geometry, solvent interactions, and electronic structure, all of which are instrumental in shaping the photophysical properties of biomimetic rotors like MeO-NAIP.

Section VII. Coupling of the ρ promoter mode with β and δ_{op}.

In our previous discussion, we uncovered a distinct, slower promoter mode ρ, which operates on a timescale of approximately 250 fs. This is in stark contrast to the well-documented 40 fs hydrogen out-of-plane wag mode, δ_{op}, observed in rPSB11@Rh and also present in the synthetic rotor MeO-NAIP. It's crucial to emphasize that this newly identified ρ mode isn't merely another manifestation of previously reported wag modes. Instead, it represents a slower ring-inversion motion that perfectly synchronizes with the reaction coordinate, resulting in a substantial increase in quantum efficiency. This mechanism goes beyond previous explanations found in synthetic rotor literature and carries direct implications for rotor engineering.

To further illustrate the distinction between the ring-puckering motion, ρ, and the COOP motion, δop (analogous to the previously reported HOOP motion in rPSB11@Rh), we refer to Figure S12. This figure provides a clear visualization of the markedly different frequencies characterizing these two modes. Specifically, ρ (orange curve) exhibits a larger oscillation period of approximately 250 fs, whereas δop (blue), which is directly related to β (black), displays a much shorter period of approximately 40 fs. Notably, the promoter ρ is intricately coupled with the motion describing the substituent wag relative to the molecule's conjugated framework β. This coupling leads to periodic changes in the amplitude of the β oscillation, occurring roughly every 200-250 fs. Consequently, these amplitude variations in β have a direct impact on the amplitude of δop, τ, and subsequently result in enhanced quantum efficiency, Φ_{iso}.

To offer visual support for the concepts discussed above, we have included accompanying movies that depict the distinct nature of these vibration modes in MeO-NAIP@gas: 1) the local 40 fs period mode β, and 2) the approximately 250 fs period ring-inversion mode ρ. See Supplementary Movie 1 and 2 for β and ρ respectively. Supplementary Movie 3 shows the synchronization of these quantities.

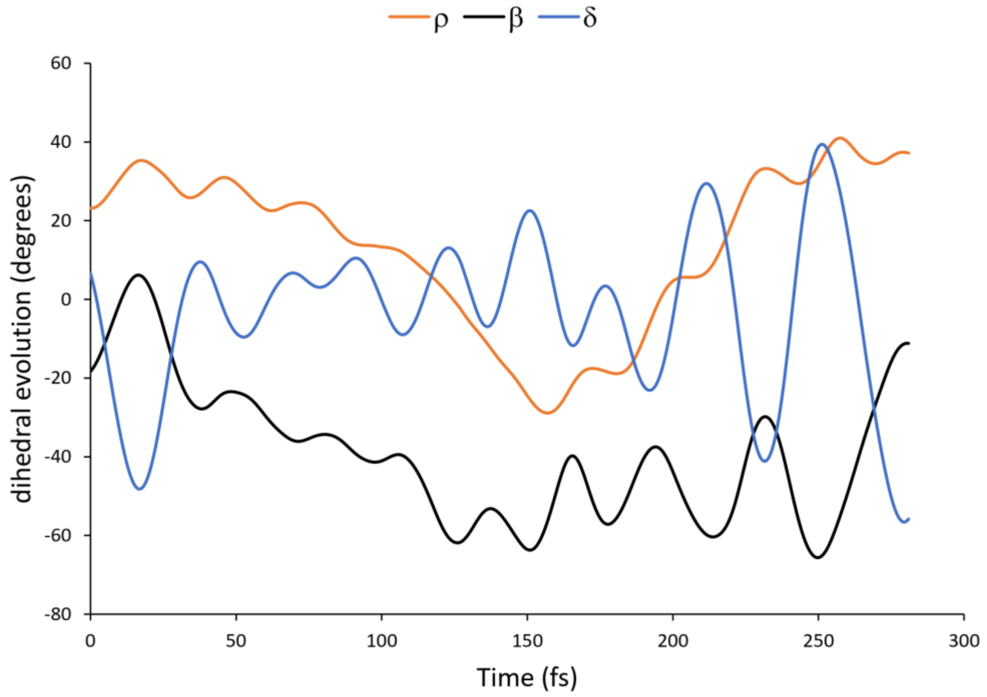

Figure S12. MeO-NAIP dihedral evolution of ρ (orange), δ_{op} (blue), and β (black) for a selected geometry in the gas-phase.

References

(1) Okada, T.; Sugihara, M.; Bondar, A. N.; Elstner, M.; Entel, P.; Buss, V. The Retinal Conformation and Its Environment in Rhodopsin in Light of a New 2.2 Å Crystal Structure. *J Mol Biol* **2004**, *342* (2), 571–583. https://doi.org/10.1016/j.jmb.2004.07.044.

(2) Schnedermann, C.; Yang, X.; Liebel, M.; Spillane, K. M.; Lugtenburg, J.; Fernández, I.; Valentini, A.; Schapiro, I.; Olivucci, M.; Kukura, P.; Mathies, R. A. Evidence for a Vibrational Phase-Dependent Isotope Effect on the Photochemistry of Vision. *Nat Chem* **2018**, *10* (4), 449–455. https://doi.org/10.1038/s41557-018-0014-y.

(3) Cornell, W. D.; Cieplak, P.; Bayly, C. I.; Gould, I. R.; Merz, K. M.; Ferguson, D. M.; Spellmeyer, D. C.; Fox, T.; Caldwell, J. W.; Kollman, P. A. A Second Generation Force Field for the Simulation of Proteins, Nucleic Acids, and Organic Molecules J . Am . Chem . Soc . 1995 , 117 , 5179−5197 . *J Am Chem Soc* **1996**, *118* (9), 2309–2309. https://doi.org/10.1021/ja955032e.

(4) Ferré, N.; Cembran, A.; Garavelli, M.; Olivucci, M. Complete-Active-Space Self-Consistent-Field/Amber Parameterization of the Lys296-Retinal-Glu113 Rhodopsin Chromophore-Counterion System. In *Theoretical Chemistry Accounts*; 2004; Vol. 112, pp 335–341. https://doi.org/10.1007/s00214-004-0593-0.

(5) Aquilante, F.; Autschbach, J.; Carlson, R. K.; Chibotaru, L. F.; Delcey, M. G.; De Vico, L.; Fdez. Galván, I.; Ferré, N.; Frutos, L. M.; Gagliardi, L.; Garavelli, M.; Giussani, A.; Hoyer, C. E.; Li Manni, G.; Lischka, H.; Ma, D.; Malmqvist, P. Å.; Müller, T.; Nenov, A.; Olivucci, M.; Pedersen, T. B.; Peng, D.; Plasser, F.; Pritchard, B.; Reiher, M.; Rivalta, I.; Schapiro, I.; Segarra-Martí, J.; Stenrup, M.; Truhlar, D. G.; Ungur, L.; Valentini, A.; Vancoillie, S.; Veryazov, V.; Vysotskiy, V. P.; Weingart, O.; Zapata, F.; Lindh, R. Molcas 8: New Capabilities for Multiconfigurational Quantum Chemical Calculations across the Periodic Table. *J Comput Chem* **2016**, *37* (5), 506–541. https://doi.org/10.1002/jcc.24221.

(6) Melaccio, F.; Olivucci, M.; Lindh, R.; Ferré, N. Unique QM/MM Potential Energy Surface Exploration Using Microiterations. *Int J Quantum Chem* **2011**, *111* (13), 3339–3346. https://doi.org/10.1002/qua.23067.

(7) Gueye, M.; Manathunga, M.; Agathangelou, D.; Orozco, Y.; Paolino, M.; Fusi, S.; Haacke, S.; Olivucci, M.; Leónard, J. Engineering the Vibrational Coherence of Vision into a Synthetic Molecular Device. *Nat Commun* **2018**, *9* (1), 313. https://doi.org/10.1038/s41467-017-02668-w.

(8) Caleman, C.; Van Maaren, P. J.; Hong, M.; Hub, J. S.; Costa, L. T.; Van Der Spoel, D. Force Field Benchmark of Organic Liquids: Density, Enthalpy of Vaporization, Heat Capacities, Surface Tension, Isothermal Compressibility, Volumetric Expansion Coefficient, and Dielectric Constant. *J Chem Theory Comput* **2012**, *8* (1), 61–74. https://doi.org/10.1021/ct200731v.

(9) Pronk, S.; Páll, S.; Schulz, R.; Larsson, P.; Bjelkmar, P.; Apostolov, R.; Shirts, M. R.; Smith, J. C.; Kasson, P. M.; Van Der Spoel, D.; Hess, B.; Lindahl, E. GROMACS 4.5: A High-Throughput and Highly Parallel Open Source Molecular Simulation Toolkit. *Bioinformatics* **2013**, *29* (7), 845–854. https://doi.org/10.1093/bioinformatics/btt055.

(10) Yang, X.; Manathunga, M.; Gozem, S.; Léonard, J.; Andruniów, T.; Olivucci, M. Quantum–Classical Simulations of Rhodopsin Reveal Excited-State Population Splitting and Its Effects on Quantum Efficiency. *Nat Chem* **2022**, *14* (4), 441–449. https://doi.org/10.1038/s41557-022-00892-6.

(11) Gueye, M.; Manathunga, M.; Agathangelou, D.; Orozco, Y.; Paolino, M.; Fusi, S.; Haacke, S.; Olivucci, M.; Leónard, J. Engineering the Vibrational Coherence of Vision into a Synthetic Molecular Device. *Nat Commun* **2018**, *9* (1), 313. https://doi.org/10.1038/s41467-017-02668-w.

(12) Paolino, M.; Giovannini, T.; Manathunga, M.; Latterini, L.; Zampini, G.; Pierron, R.; Léonard, J.; Fusi, S.; Giorgi, G.; Giuliani, G.; Cappelli, A.; Cappelli, C.; Olivucci, M. On the Transition from a Biomimetic Molecular Switch to a Rotary Molecular Motor. *Journal of Physical Chemistry Letters* **2021**, *12* (16), 3875–3884. https://doi.org/10.1021/ACS.JPCLETT.1C00526/SUPPL_FILE/JZ1C00526_SI_001.PDF.

(13) Granucci, G.; Persico, M. Critical Appraisal of the Fewest Switches Algorithm for Surface Hopping. *Journal of Chemical Physics* **2007**, *126* (13). https://doi.org/10.1063/1.2715585.

(14) Tully, J. C. Molecular Dynamics with Electronic Transitions. *J Chem Phys* **1990**, *93* (2), 1061–1071. https://doi.org/10.1063/1.459170.

(15) Marsili, E.; Olivucci, M.; Lauvergnat, D.; Agostini, F. Quantum and Quantum-Classical Studies of the Photoisomerization of a Retinal Chromophore Model. *J Chem Theory Comput* **2020**, *16* (10), 6032–6048. https://doi.org/10.1021/ACS.JCTC.0C00679/SUPPL_FILE/CT0C00679_SI_002.MP4.

(16) Manathunga, M.; Yang, X.; Luk, H. L.; Gozem, S.; Frutos, L. M.; Valentini, A.; Ferrè, N.; Olivucci, M. Probing the Photodynamics of Rhodopsins with Reduced Retinal Chromophores. *J Chem Theory Comput* **2016**, *12* (2), 839–850. https://doi.org/10.1021/acs.jctc.5b00945.
(17) Johnson, P. J. M.; Halpin, A.; Morizumi, T.; Prokhorenko, V. I.; Ernst, O. P.; Miller, R. J. D. Local Vibrational Coherences Drive the Primary Photochemistry of Vision. *Nat Chem* **2015**, *7* (12), 980–986. https://doi.org/10.1038/nchem.2398.
(18) Polli, D.; Altoè, P.; Weingart, O.; Spillane, K. M.; Manzoni, C.; Brida, D.; Tomasello, G.; Orlandi, G.; Kukura, P.; Mathies, R. A.; Garavelli, M.; Cerullo, G. Conical Intersection Dynamics of the Primary Photoisomerization Event in Vision. *Nature* **2010**, *467* (7314), 440–443. https://doi.org/10.1038/nature09346.
(19) Dartnall, H. J. A. The Photosensitivities of Visual Pigments in the Presence of Hydroxylamine. *Vision Res* **1968**, *8* (4), 339–358. https://doi.org/10.1016/0042-6989(68)90104-1.
(20) Lumento, F.; Zanirato, V.; Fusi, S.; Busi, E.; Latterini, L.; Elisei, F.; Sinicropi, A.; Andruniów, T.; Ferré, N.; Basosi, R.; Olivucci, M. Quantum Chemical Modeling and Preparation of a Biomimetic Photochemical Switch. *Angewandte Chemie - International Edition* **2007**, *46* (3), 414–420. https://doi.org/10.1002/anie.200602915.
(21) Gueye, M.; Paolino, M.; Gindensperger, E.; Haacke, S.; Olivucci, M.; Léonard, J. Vibrational Coherence and Quantum Yield of Retinal-Chromophore-Inspired Molecular Switches†. *Faraday Discuss* **2019**, *221*, 299–321. https://doi.org/10.1039/c9fd00062c.
(22) Sinicropi, A.; Martin, E.; Ryazantsev, M.; Helbing, J.; Briand, J.; Sharma, D.; Léonard, J.; Haacke, S.; Cannizzo, A.; Chergui, M.; Zanirato, V.; Fusi, S.; Santoro, F.; Basosi, R.; Ferré, N.; Olivucci, M. An Artificial Molecular Switch That Mimics the Visual Pigment and Completes Its Photocycle in Picoseconds. *Proc Natl Acad Sci U S A* **2008**, *105* (46), 17642–17647. https://doi.org/10.1073/pnas.0802376105.
