# Supplementary material for: Comparative quantum-classical dynamics of natural and synthetic molecular rotors show how vibrational synchronization modulates the photoisomerization quantum efficiency
Source: Nat Commun. 2024 Apr 25;15:3499. doi: 10.1038/s41467-024-47477-0 (PMC11045841; doi:10.1038/s41467-024-47477-0)
Supplement: Supplementary file 3 — Description of Supplementary Files [file 41467_2024_47477_MOESM3_ESM.pdf]

Description of Additional Supplementary Files

File Name: Supplementary Movie 1

Description: This movie highlights MeO-NAIP's wag substituent motion (β) with a 40-fs oscillation period, analogous to the hydrogen out-of-plane wag mode (β) observed in rPSB11@Rh, crucial for quantum efficiency enhancement. However, this motion in MeO-NAIP does not independently modulate quantum efficiency, illustrating the intricate factors controlling the efficiency of the molecular rotor.

File Name: Supplementary Movie 2

Description: This movie showcases the slower ring-inversion motion (ρ) in MeO-NAIP, with a 250-fs oscillation period, which appears to synchronize with the reaction coordinate to boost quantum efficiency significantly. This insight advances rotor engineering beyond previous models, emphasizing the ρ mode's critical role in enhancing efficiency.

File Name: Supplementary Movie 3

Description: This movie reveals the dynamic coupling between the ring-inversion (ρ) and wag substituent (β) motions in MeO-NAIP, leading to periodic β oscillation amplitude changes every 200-250 fs. These variations influence quantum efficiency, showcasing the ρ mode's pivotal role as a promoter in efficiency enhancement.

The Supplementary Data 1-5 below are also made available in the following Zenodo repository: Link checked: https://zenodo.org/records/10884008

File Name: Supplementary Data 1

Description: This file provides XYZ coordinates for approximately two hundred trajectories related to the population dynamics of the rPSB11@Rh system (timestep 1 fs).

File Name: Supplementary Data 2

Description: This file provides XYZ coordinates for approximately two hundred trajectories related to the population dynamics of the rPSB11 isolated system (timestep 1 fs).

File Name: Supplementary Data 3

Description: This file provides XYZ coordinates for approximately two hundred trajectories related to the population dynamics of the NAIP@MeOH system (timestep 1 fs).

File Name: Supplementary Data 4

Description: This file provides XYZ coordinates for approximately two hundred trajectories related to the population dynamics of the NAIP isolated system (timestep 1 fs).

File Name: Supplementary Data 5

Description: This file provides XYZ coordinates for approximately one hundred trajectories related to the population dynamics of the NAIP@DMSO system (timestep 1 fs).
