# Supplementary material for: Comparative quantum-classical dynamics of natural and synthetic molecular rotors show how vibrational synchronization modulates the photoisomerization quantum efficiency
Source: Nat Commun. 2024 Apr 25;15:3499. doi: 10.1038/s41467-024-47477-0 (PMC11045841; doi:10.1038/s41467-024-47477-0)
Supplement: Supplementary file 13 — Source Data Inventory [file 41467_2024_47477_MOESM13_ESM.pdf]

The Source Data File provided includes:

- ChemDraw files (one zipped file)
- Original Figures (one zipped file)
- All raw data for figures (one zipped file) in the main manuscript and Supplementary Information, organized as follows:
  - Excel file with plot data for the Figures.
  - Separate zipped file containing the trajectory data used for generating the trajectory plots.
